# Rheological and Micro-Rheological Properties of Chicory Inulin Gels †

**DOI:** 10.3390/gels10030171

**Published:** 2024-02-28

**Authors:** Jingyuan Xu, James A. Kenar

**Affiliations:** 1 Plant Polymer Research, National Center for Agricultural Utilization Research, Agricultural Research Service, US Department of Agriculture, 1815 North University Street, Peoria, IL 61604, USA; 2 Functional Food Research, National Center for Agricultural Utilization Research, Agricultural Research Service, US Department of Agriculture, 1815 North University Street, Peoria, IL 61604, USA

**Keywords:** diffusion wave spectroscopy (DWS), inulin, micro-rheology, rheology

## Abstract

As a soluble fiber, inulin is present in many plants and has many applications in food and non-food products. In this work, we investigated the rheological properties of inulin dispersions at seven concentrations. The linear viscoelastic properties of inulin were determined using a conventional mechanical rheometer. At 25 wt%, inulin exhibited fluid-like viscoelastic liquid behavior. However, when concentrations were ≥27.5 wt%, inulin exhibited gel-like viscoelastic properties. The viscoelastic properties (moduli and viscosities) increased with increasing inulin concentration. The high-frequency linear rheological properties of inulin were also investigated using the modern light scattering technique, diffusion wave spectroscopy (DWS). The diffusion wave spectroscopy (DWS) measurements showed the amplitude of complex moduli (|G*(ω)|) of inulin gels (≥27.5 wt%) to be proportional to ½ power law of the frequency, which suggests inulin gels behave similarly to flexible polymers. The non-linear steady shear experiments demonstrated that inulin exhibited shear-thinning behavior that was well fitted by a power law constitutive model. The trend of the power law exponent from the experiments indicated that the shear-thinning extent for inulin was greater as the inulin concentration increased. The results of this work indicated that the properties of inulin gel can be manipulated by altering its concentration. Therefore, the desired inulin product can be designed accordingly. These results can be used to direct further food and non-food applications, such as wound healing materials for inulin gels.

## 1. Introduction

Inulin is an inexpensive, naturally occurring, water-soluble carbohydrate. It is usually isolated from various parts, including fruits, roots, or rhizomes, of more than 36,000 plant species, such as wheat, oats, rye, barley, onion, bananas, tomato, garlic, asparagus, chicory, artichokes, etc. [1,2]. Usually, inulin is produced from chicory root because chicory root has the highest content of inulin (65–79%) among all the sources [1,2]. In recent years, the extraction, isolation, purification, and characterization of inulin have received a lot of attention due to its widespread availability. Many studies have been developed for optimizing the methods in order to improve inulin purity and production from the plants [1,2]. The production process of inulin from chicory root has three basic steps: (1) extraction, (2) separation of purified compound (purification), and (3) drying [1,2]. Inulin can also be synthesized from various enzymatic processes using bacterial and fungal species such as *Bacilli*, *Pseudomonas*, *Aspergillus sydowi*, and *Streptococci*. The inulin obtained from natural sources has a shorter chain length and a lower molecular weight (LMW) compared to thosesynthesized [1,2]. The low-molecular-weight natural inulin (relative short chain) is sweet, but the synthetic one (high molecular weight and relative long chain) is tasteless. Because of this feature, many food industries are using low-molecular-weight natural inulin as a low-calorie food and natural sweetening agent [1,2]. Inulin is a heterogeneous blend of polymers composed of fructose molecules linked through β(2,1) bonds and having a glucose end group. Because of the β(2,1) linkages, inulin cannot be digested by human digestive enzymes but can be fermented and hydrolyzed by colon bacteria. Inulin is classified as a soluble dietary fiber with prebiotic properties and is beneficial to humans in reducing blood lipid and sugar levels, weight loss, enhancing absorption of minerals and vitamins, and stimulating the growth of beneficial gut bacteria [3]. In addition, inulin has shown some potential therapeutic benefits by having antioxidant activities, enhancing absorption of calcium, reducing the risk of some cancers, helping in the removal of constipation, and improving the anti-biofilm behavior of some antimicrobial molecules, etc. [1,2,3]. For the delivery of therapeutics, inulin-based carriers have been used as polysaccharide-coated nanocarriers, hydrogels, lipid–polysaccharide conjugates, drug–polysaccharide conjugates, polysaccharide–carbon nanomaterial conjugates, polymeric–polysaccharide/copolymer conjugates, etc. [1,2,3]. Inulin has also been used as a drug stabilizer in pharmaceutical formulations, as a modifier and/or carrier for drug delivery, and as a diagnostic/therapy agent for diseases [4]. There is a report recently indicating that inulin-based films can be used as wound healing materials [5]. Wound healing materials would be biocompatible with the human body and have a soft consistency with a high water content. And wound healing gels provide mechanical support and adhesion sites for cells in the living body and give mechanical strength and structural integrity to tissues and organisms [6,7,8]. Inulin’s biological nature, gel formation with high water content, and soft consistency make inulin gel a good candidate for wound healing material [5]. Moreover, inulin has many applications in the food industry as a fat replacer and a substitute for starch [9].

When inulin is dispersed into water, it forms a white creamy three-dimensional gel network composed of sub-micron particles [5,10]. The resulting inulin gels are stable in acidic and high-temperature conditions and provide interesting opportunities to modify rheological properties in food and nonfood applications. Thus, understanding the rheological properties of inulin gels is critical to developing processing techniques and new products containing inulin. Some research concerning the rheological properties of products using inulin has been reported. For example, Pitarreso et al. examined the rheological properties of methacrylated inulin derivative hydrogels and their possible use in drug delivery applications [3]. Tárrega et al. studied the rheological properties of dairy desserts enriched in inulin with short, medium, and long chain lengths [11]. Kumar and coworkers explored the solution rheology of inulin at very low concentrations over different temperatures [12]. Kim and Wang examined inulin gels produced by heating and cooling inulin solutions and found inulin hydrolysis during heating to be an important factor in gel formation [13]. Although some rheological characteristics of inulin gels are known, further understanding of inulin gels is warranted in order to enable further applications as well as novel applications of inulin, such as wound healing gels. In this work, the rheological and micro-rheological properties of inulin gels prepared over a range of concentrations were explored using conventional rheology and diffusion wave spectroscopy (DSW) techniques. The resulting rheological properties of these inulin gels provide valuable information applicable to developing new uses for inulin. The micro-rheological properties of inulin gels will give us more insight into the basic physical behaviors of inulin polymers. The results of this research are useful and important to identify new food and non-food applications of inulin.

## 2. Results and Discussion

**Figure 1:**
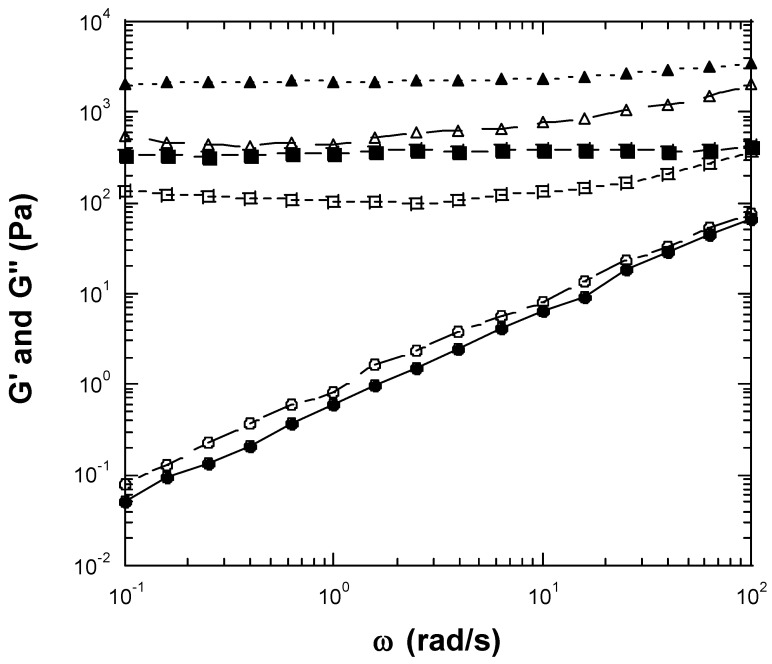
Linear viscoelastic properties measured by conventional mechanical rheometer for 25, 27.5, and 30 wt% inulin. Storage modulus (G′) or loss modulus (G″) vs. frequency at 25 °C with 0.08% strain. G′ is indicated using filled symbols, while G″ is indicated using opened symbols. (●, ○): 25 wt% inulin. (■, □): 27.5 wt% inulin gel. (▲, △): 30 wt% inulin gel.

**Figure 2:**
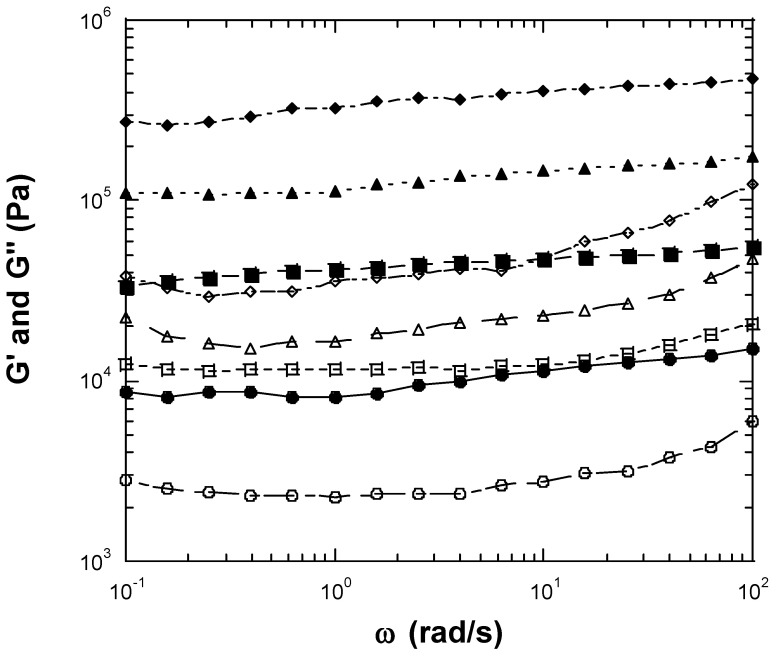
Linear viscoelastic properties measured by conventional mechanical rheometer for 32.5, 35, 37.5%, and 40 wt% inulin gels. Storage modulus (G′) or loss modulus (G″) vs. frequency at 25 °C with 0.08% strain. G′ values are expressed with filled symbols, while G″ values are expressed with unfilled symbols. (●, ○): 32.5 wt% inulin gel. (■, □): 35 wt% inulin gel. (▲, △): 37.5 wt% inulin gel. (◆, ◇): 40 wt% inulin gel.

**Figure 3:**
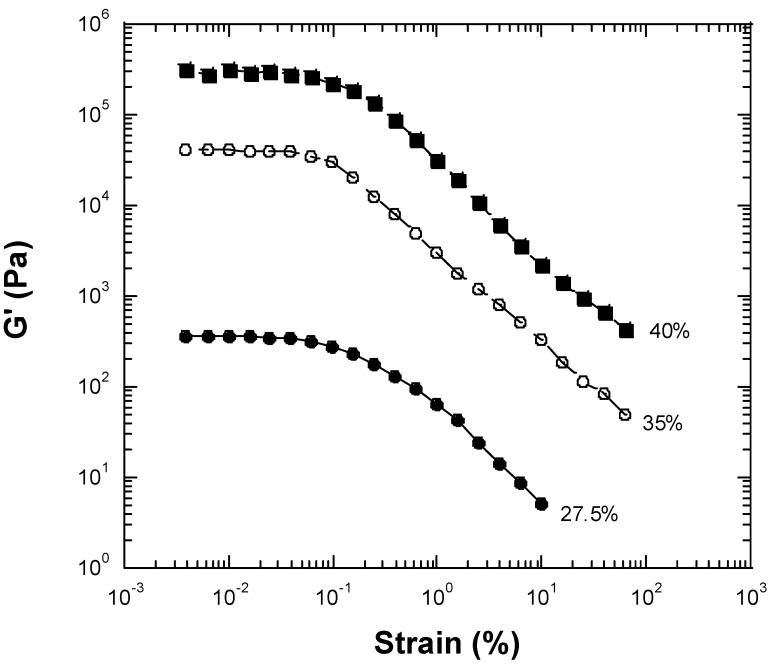
Results of strain sweep experiments for inulin gels measured using a conventional mechanical rheometer operating at 1 rad/s frequency and a temperature of 25 °C. (●): 27.5 wt% inulin gel. (○): 35 wt% inulin gel. (■): 40 wt% inulin gel.

We examined a wide range of inulin concentrations and selected a range of concentrations that included fluid-like behavior and gel-like behavior. We measured seven concentrations of inulin, from a relatively low concentration of 25 wt% to a relatively high concentration of 40 wt%. When high concentrations of inulin were dissolved in water, a creamy gel-like material resulted. The linear rheological properties of dynamic frequency sweep of the inulin solutions are shown in Figure 1 and Figure 2. At an inulin concentration of 25 wt%, both storage (elastic) moduli (G′) and loss moduli (G″) exhibited straight lines with a slope near unity, and G″s were greater than G′s. G′ at 1 rad/s was 0.59 Pa (Figure 1). The phase shifts were in the range of 49.5°–60.4°. The straight line moduli followed a power law of one and indicated that the sample had liquid fluid-like viscoelastic behavior at concentrations of less than 25 wt% [14,15]. Therefore, at dilute concentrations (25 wt% or below), the chain–chain interactions between inulin polymers were not sufficient to form a gel-like network. As a result, fluid or liquid-like properties predominated. At an inulin concentration of 27.5 wt%, both G′ and G″ were greater than those for 25 wt% inulin, demonstrating higher viscoelastic properties. Both G′ and G″ of the 27.5 wt% inulin sample showed frequency independence, with G′s being larger than G″s. G′ at 1 rad/s was 355.7 Pa (Figure 1). The phase shifts of 27.5 wt% inulin ranged between 14.8° and 41.5°, becoming much smaller than those of 25 wt% inulin. This result suggests the 27.5 wt% inulin solution exhibits gel-like viscoelastic solid behavior. Within a narrow concentration range between 25 wt% and 27.7 wt%, the inulin solution shifts from liquid-like viscoelastic behavior to gel-like viscoelastic solid behavior. With increasing inulin concentration, the gel-like viscoelastic behavior becomes more prominent, as evidenced by the higher moduli, much more flattened-shape moduli responses, and smaller phase shifts (Figure 1 and Figure 2). The observed flattening or frequency-independent nature of the moduli curves are typical for gels and viscoelastic solids [14,15,16]. At 30 wt% inulin, a gel is produced with G′ at 1 rad/s, which can be considered a plateau modulus, being 2.2 × 10^3^ Pa (Figure 1). The phase shifts of the 30% inulin gel ranged from 13.7° to 21.3°. For the 32.5 wt% inulin gel, the G′ at 1 rad/s increased to 8.2 × 10^3^ Pa (Figure 2). The phase shifts of the 32.5 wt% inulin gel were in the range of 13.3°–21.3°. For 35 wt% inulin gel, the G′ at 1 rad/s increased to 4.2 × 10^4^ Pa (Figure 2). The phase shifts of the 35 wt% inulin gel decreased into the range of 13.3°–20.6°. For the 37.5 wt% inulin gel, the G′ at 1 rad/s increased to 1.1 × 10^5^ Pa (Figure 2). The phase shifts of the 37.5 wt% inulin gel were in the range of 7.9°–15.4°. For 40 wt% inulin gel, the G′ at 1 rad/s jumped to 3.2 × 10^5^ Pa (Figure 2). The phase shifts of the 40 wt% inulin gel dropped in the range of 7.2°–14.7°. As a reference, G′ and the phase shift for synthetic rubber, which is a viscoelastic solid, are about 10^7^ Pa and 11°, respectively. Thus, the inulin solutions formed gels above 27.5 wt%, and the rheological properties (G′ and G″) of the gels increased with increasing inulin concentration. Figure 3 presents results from strain sweep experiments of three inulin gels corresponding to inulin concentrations of 27.5, 35, and 40 wt%. According to the above results, inulin molecule–molecule interactions should be very weak at lower concentrations of ≤25 wt%, and a gel cannot be established at these dilute concentrations. However, at higher concentrations of inulin (≥27.5 wt%), the inulin chain–chain interactions and entanglements become stronger and stronger with increasing concentration, and gels would be formed. The linear range of all measured inulin gels (27.5 wt%, 30 wt%, 32.5 wt%, 35 wt%, 37.5 wt%, and 40 wt% inulin gels) was less than 0.1% strain (Figure 3 just shows 27.5 wt%, 35 wt%, and 40 wt% inulin’s strain sweep data), which was rather small. The gels exhibiting a small linear range (less than 20%) suggest the networks are so-called ‘weak’ gels that are likely to be disrupted under shear or other perturbations [14,16]. In contrast, a ‘strong’ gel can typically withstand perturbations and deformations to a strain of 20% or higher. Therefore, the strain sweep tests indicated that inulin gels are ‘weak’ gels instead of ‘strong’ gels.

**Figure 4:**
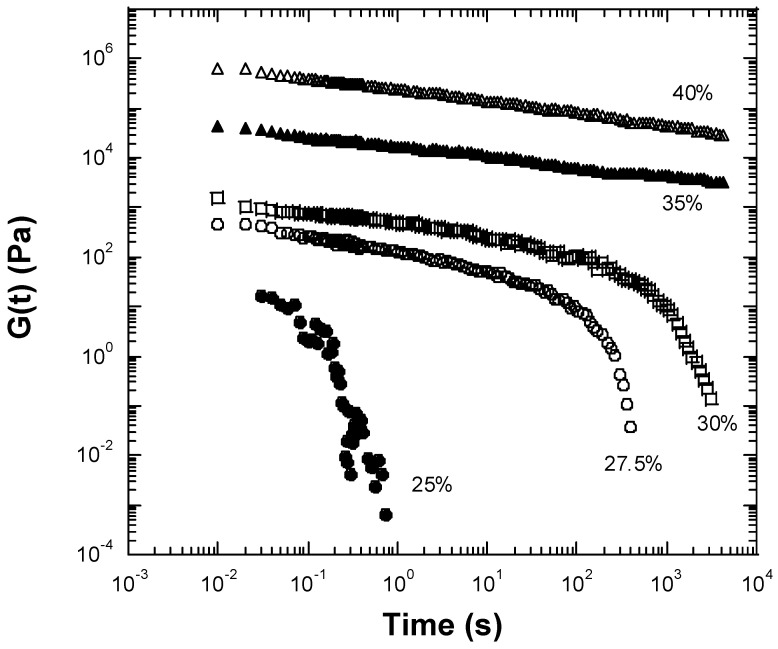
Stress relaxation experiments measured using a conventional mechanical rheometer for the inulin samples after being subjected to a 0.08% strain at 25 °C. (●): 25 wt% inulin. (○): 27.5 wt% inulin gel. (□): 30 wt% inulin gel. (▲): 35 wt% inulin gel. (△): 40 wt% inulin gel.

A gel is normally formed through the chemical (covalent) or physical cross-linking of polymer chains into a three-dimensional network. To investigate whether the prepared inulin gels were chemically or physically cross-linked networks, stress relaxation experiments were conducted (Figure 4). As seen in the figure, the 25 wt% inulin sample was immediately fully relaxed within a second after an initial step change in shear strain within the linear range. This result indicates no network or gel formation for the 25 wt% inulin, which is in agreement with the above fact that 25 wt% inulin exhibited fluid-like behavior rather than gel-like behavior. In contrast, the 27.5 wt% inulin sample relaxed more slowly and fully relaxed after approximately 300 s (Figure 4). As the inulin concentration increased to 30 wt%, the gel took 1000 s to fully relax (Figure 4). Finally, the 35 wt% and 40 wt% inulin gels relaxed very slowly and did not fully relax even after 3000 s (Figure 4). These results demonstrate that the inulin gels are composed of three-dimensional, physically crosslinked networks as opposed to being chemically crosslinked. If the gels were chemically crosslinked, no relaxation would be observed, and the relaxation time would be infinite. Thus, based on these relaxation time results, the inulin gels prepared in this study consist of physically crosslinked networks due to chain–chain interactions and entanglements that become more prevalent as the concentration of inulin increases.

**Figure 5:**
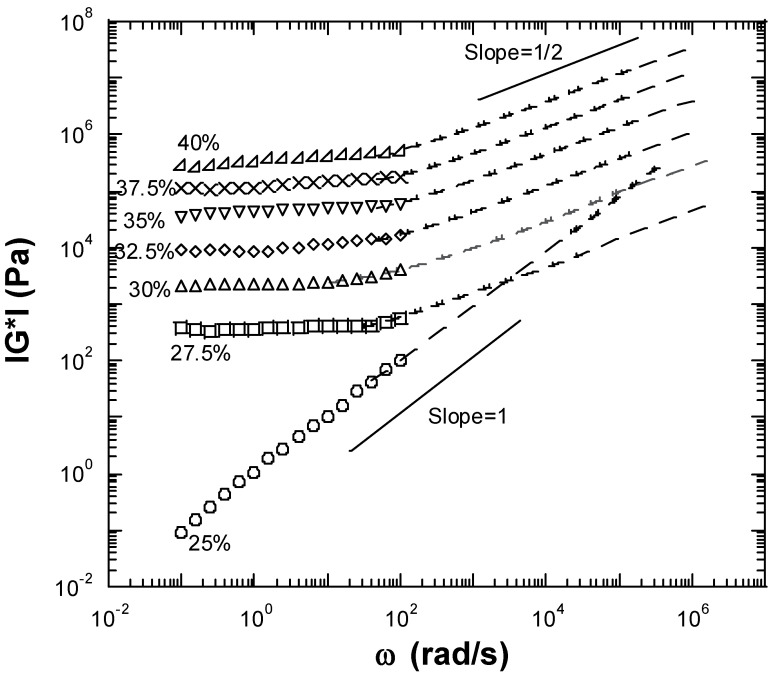
The |G*| = (G′^2^ + G″^2^)^1/2^ of the 25%, 27.5%, 30%, 32.5%, 35%, 37.5%, and 40 wt% inulin samples measured by a conventional mechanical rheometer (symbols) and DWS (dashed lines) at 25 °C. The two straight lines show slopes of 1 and 1/2, respectively.

The high-frequency range viscoelastic properties show the early dynamics of relaxation of the polymer materials. However, it is nearly impossible to measure a sample’s high-frequency viscoelastic properties by conventional mechanical rheometry at frequencies greater than 200 rad/s. Diffusion wave spectroscopy (DWS) provides an opportunity to detect the high-frequency-range viscoelastic properties of polymers. Additionally, it is noninvasive, allowing rheological properties to be measured without disturbing the gel structure, making it suitable to study fragile systems. Figure 5 shows the amplitude of the viscoelastic moduli (|G*(ω)|) measured for the seven inulin concentrations examined by both the mechanical rheometer and DWS. The observed overlap in the curves obtained with the mechanical rheometer and the DWS instrument demonstrate excellent consistency between the two techniques (Figure 5). For the 25 wt% inulin sample, the complex moduli (|G*(ω)|) as a function of frequency measured by both the mechanical rheometer and DWS was a straight line following the power law of unity (Figure 5). This result supported the fact that 25 wt% inulin was a fluid-like viscoelastic liquid and did not form a gel. The complex moduli (|G*(ω)|) at high frequencies for all measured inulin gels (27.5–40 wt%) were large, implying the total resistance of the inulin gels to the external force was very high within an extremely short time. The high-frequency |G*(ω)| for all measured inulin gels (27.5–40 wt%) presented the same behavior as |G*(ω)| ∝ ω^1/2^ (Figure 5). The high-frequency behaviors of |G*(ω)| can be predicted based on theoretical models. One model depicts flexible polymers as displaying behavior consistent with the following relationship at high frequencies: |G*(ω)| ∝ ω^α^, with α = 1/2, which is in excellent agreement with our DWS measurements for the inulin gels (Figure 5) [15,17,18]. Therefore, the DWS results for inulin gels (≥27.5 wt%) suggest that they exhibit flexible polymer behavior and provide us with additional insight into the characteristics of these gels. The complex moduli (|G*(ω)|) represent the material’s total resistance to the external force or strain. The great value of (|G*(ω)|) at high frequencies for inulin gels suggested that the inulin gels would strongly resist the very high-frequency external disturbance (external force within a very short time).

**Figure 6:**
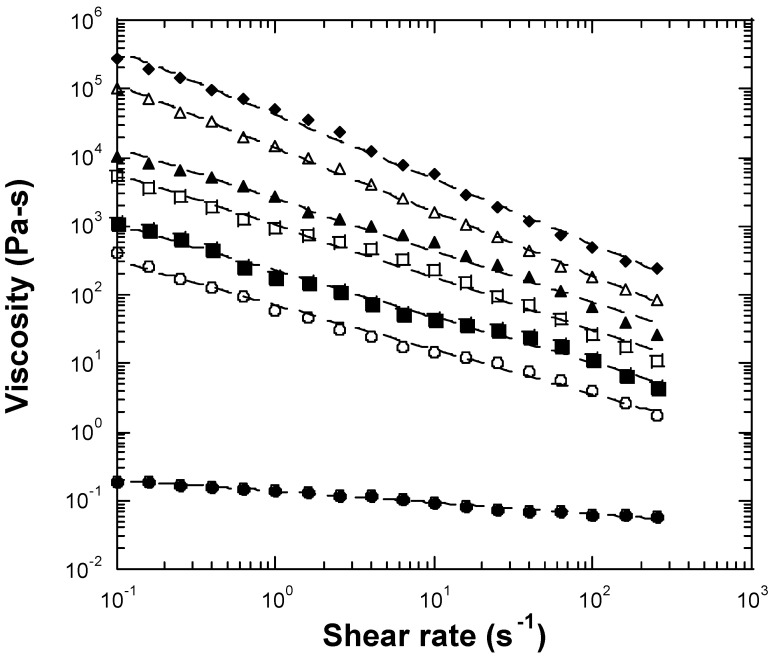
Plots of viscosity vs. shear rate measured using a conventional mechanical rheometer for the 25 wt% (●), 27.5 wt% (○), 30 wt% (■), 32.5 wt% (□), 35 wt% (▲), 37.5 wt% (△), and 40 wt% (◆) inulin dispersions at 25 °C. Dashed lines are fitted results by the power law model (Equation (1)).

To better understand processing behavior, the non-linear steady shear viscoelastic properties of the inulin samples were studied by mechanical rheometry. The non-linear viscoelastic behavior of the samples was shown to be dependent on inulin concentration (Figure 6), with viscosities increasing with increasing concentration as expected. All seven samples of varying concentrations of inulin displayed shear-thinning behavior over the measured shear rates (Figure 6). Shear-thinning rheological behavior can be characterized by a power law constitutive equation [14,19]. The power law equation can be expressed as

η = Kγ^n−1^ (1)

where η is the shear viscosity, K is the front factor, γ is the shear rate, and n is the power law exponent. The power law exponent represents shear-thinning extent, with a smaller exponent indicating greater shear-thinning behavior. A Newtonian fluid is typically a viscous fluid or liquid with the power law exponent n = 1 and displays no shear-thinning behavior. In contrast, many polymers show non-Newtonian, shear-thinning behavior with the power law exponent n < 1. We used Equation (1) to fit shear-thinning viscosity data for the inulin samples investigated (Figure 6). The experimental data showed a good fit to the power law constitutive equation (Figure 6), and the fitting results are summarized in Table 1. The 25 wt% inulin sample showed properties similar to those of a Newtonian fluid and a slight shear-thinning behavior with an exponent of 0.83 (Figure 6), consistent with the fluid-like performance found at linear viscoelastic measurements. At higher concentrations (≥27.5 wt%), the inulin gels exhibited greater shear-thinning behavior, as evidenced by a diminishing exponent with increasing inulin concentration (Figure 6 and Table 1). The exponents for the 27.5, 30, 32.5, 35, 37.5, and 40 wt% inulin gels were 0.35, 0.33, 0.25, 0.24, 0.07, and 0.06, respectively (Table 1). The majority of the processing shear rates are within the range of 1 s^−1^ to 200 s^−1^. The above results of non-linear shear viscosity for the inulin samples can be useful to understand appropriate processing conditions and properties for aqueous inulin dispersions.

The linear and non-linear rheological properties of inulin in this study exhibited similar behaviors to those of many wound healing gels [8]. Thus, inulin can be a good candidate for wound healing material due to not only its biodegradable nature but also its properties. In addition, this work showed that the properties of inulin can be manipulated by varying its concentrations. Therefore, inulin gels can be designed according to their usage, such as cosmetic gels, food coating materials, and wound healing materials.

## 3. Conclusions

In this work, we studied the rheological properties of aqueous inulin gels using a conventional mechanical rheometer as well as the micro-rheological properties using diffusion wave spectroscopy (DWS). A relatively narrow concentration (25 to 27.5 wt%) of inulin induced a transition of the linear rheological properties. At inulin concentrations of 25 wt% or less, fluid-like viscoelastic behavior—both elastic and loss moduli—followed a power law straight line with a slope of one. However, at inulin concentrations of 27.5 wt% and above, gels formed, and gel-like viscoelastic solid properties were observed to have greater viscoelasticity as inulin concentration increased. At relative dilute concentrations of 25 wt% or less, the inulin chain–chain interactions are very weak, and there is no gel formed. When concentration increases, the inulin molecule–molecule interactions and entanglement become stronger and stronger at 27.5 wt% and above, and a gel would be established. The small linear range of viscoelastic property results of strain sweep testing for inulin gels advocated that inulin gels are so-called ‘weak’ gels rather than ‘strong’ gels. The stress relaxation experiments for inulin gels recommended that inulin gels be physical gels instead of chemical gels. Diffusion wave spectroscopy (DWS) allowed for the measurement of the high-frequency behavior of linear rheological properties for aqueous inulin dispersions. The high-frequency amplitude of complex moduli curves for inulin gels (≥27.5 wt%) exhibited a power law behavior with frequency with a slope of ½, that is, |G*| ∝ ω^1/2^, which signifies flexible polymer behavior. The non-linear rheological properties of all measured inulin dispersions displayed shear-thinning behavior, with higher viscosity and shear-thinning properties as inulin concentration increased. The results of this work give us new insight into inulin gels and will be useful for developing new applications such as wound healing materials for inulin.

### Future Directions

In this work, we studied the rheological properties of inulin gels at room temperature. And this work indicated that inulin gels can be designed or manipulated by altering concentration according to their usage, such as wound healing dressings. Wound healing is a dynamic process. The performance of wound care requires a dressing change as healing progresses. It is widely accepted that a warm and moist environment encourages rapid healing, and most modern wound healing products are designed to provide these conditions. Fluid balance in burn injuries is also very important since heavy loss of water from the body by exudation and evaporation may lead to a fall in body temperature and an increase in metabolic rate. In order to use inulin-based gels as wound care products, the properties of inulin gels for wound healing dressings should be further studied, such as rheology behavior and water holding at warm temperatures (body temperature 37 °C and above). Besides these, wound care dressings should have certain other properties, like ease of application and removal and proper adherence, so that there will not be any area of non-adherence left to create fluid-filled pockets for the proliferation of bacteria. Thus, inulin gel properties such as ease of application and removal and proper adherence should also be tested if using inulin gels as wound care materials.

## 4. Materials and Methods

### 4.1. Material

The inulin from chicory (white powder) was purchased from Sigma-Aldrich (St. Louis, MO, USA). The fructose/glucose ratio of this inulin was ≥20:1.

### 4.2. Inulin Gel Preparation

The chicory inulin powder was thoroughly mixed with deionized water at the desired concentration. The solution was heated to 80 °C with stirring to dissolve the powder. Then, the sample was cooled down to room temperature. Seven concentrations of inulin gels were prepared (25, 27.5, 30, 32.5, 35, 37.5, and 40 wt%). The prepared inulin gels were kept at 4 °C and used within two days.

### 4.3. Rheological Measurements Using a Mechanical Rheometer

Rheological properties measured with a mechanical rheometer were conducted using a strain-controlled Rheometric ARES rheometer (TA Instruments, New Castle, DE, USA) equipped with a 25 mm (for higher concentrations of inulin gels) or 50 mm diameter (for lower concentrations of inulin) parallel-plate fixture [20]. A water circulation system kept the sample at 25 ± 0.1 °C during analyses. Mineral oil was used to seal the plates’ edges to retain sample moisture. The sample’s viscoelasticity linear range was initially determined by conducting a strain sweep test. Subsequently, a linear strain was adopted for the other dynamic oscillatory experiments using new samples. The linear dynamic oscillatory experiments provided the sample’s elastic/storage (G′—storage) and loss/viscous (G″—dissipative) moduli. G′ is the elastic feature of the material, while G″ is the viscous feature of the sample. The magnitude of the complex modulus, (|G*(ω)| = (G′^2^ + G″^2^)^1/2^), is characterized as the total resistance to deformation of the material [17]. The phase shift or phase angle (δ) is defined by δ = tan^−1^ (G″/G′) and indicates whether a material is solid with perfect elasticity (δ = 0), liquid with pure viscose dissipation (δ = 90°), or something in between. Stress relaxation experiments were carried out in the linear viscoelastic range. These experiments measured the stress relaxation over time after the sample was subjected to a step change in strain. Non-linear rheological studies were conducted using the same ARES instrument with the same geometry described above. The steady shear measurements were conducted with the shear rate increased stepwise in the shear rate range of 0.1–300 s^−1^. All linear and non-linear rheological experiments for each sample were repeated at least three times using different samples to ensure the relative errors were within ±8%.

### 4.4. Micro-Rheology Measurements Using Diffusion Wave Spectroscopy (DWS)

The DWS is a modern light scattering technique to measure the Brownian movement of microspheres imbedded into polymer samples [17]. Basically, a laser beam is incident to a cuvette holding the sample containing the optical microbead probes and the scattered light collected by a photon detector. The outputs of the photon detector are directed into a correlator to determine the autocorrelation function. Then, the autocorrelation function with time is determined by the correlator coupled to the photon detector. The mean square displacement (MSD) of the probing beads can be extracted using a root-search algorithm from the autocorrelation function. The polymer sample’s rheological behavior (|G*(ω)|) can be calculated from the mean square displacement (MSD) using the Stokes-Einstein equation [17]. Briefly, the local modulus of a viscoelastic polymer solution and the mean square displacement of a microsphere suspended in that fluid are related as follows:

(2) $$\tilde{G}(s)=\frac{s}{6\pi a}\left[\frac{6k_{B}T}{s^{2}\langle ∆{\tilde{r}}^{2}\left(s\right)\rangle }-ms\right]\approx \frac{k_{B}T}{\pi as\langle ∆{\tilde{r}}^{2}\left(s\right)\rangle }$$

where $k_{B}$ is Boltzmann’s constant, T is the temperature of the sample, a represents the radius of the microsphere, m is its mass, s represents the Laplace frequency, and ~ is the unilateral Laplace transformation, which is defined as

(3) $$\tilde{X}(s)\equiv L\left[X(t)\right]\equiv \int_{0}^{\infty }X\left(t\right)\exp\left(-st\right)dt$$

Equation (2) relates the unilateral Laplace transform of the stress relaxation modulus $G_{r}\left(t\right),\tilde{G}(s)\equiv s\tilde{G_{r}}(s)$ to the Laplace transform of the mean-square displacement $\langle ∆{\tilde{r}}^{2}\left(t\right)\rangle$. In Equation (2), the inertia term (the second term in the brackets) can be neglected because of its corresponding to very high frequencies. The knowledge of (s) is sufficient to characterize the viscoelastic behavior of a polymer solution. However, in order to present the optical measurements in a more familiar fashion, the analytic continuation between the real function (s) and the complex function $G^{*}\equiv G^{\prime }\left(\omega \right)+iG^{\prime \prime }(\omega )$ is used. One can extract storage and loss moduli by replacing s with iω in $\tilde{G}$(*s*) and by extracting real and imaginary parts from the resulting imaginary function $\tilde{G}$(*s* = *iω*), respectively. The DWS measurements for the inulin gels were conducted using a DWS RheoLab II instrument (LS Instruments AG, Switzerland). The 450 nm diameter polystyrene microspheres (0.5%) were gently mixed into the inulin gel. The chamber holding the cuvette in the DWS RheoLab II was controlled at 25 ± 0.1 °C. The added polystyrene microspheres did not affect the viscoelastic moduli and viscosity of the inulin measured with conventional rheological measurements. All DWS measurements for each sample were repeated at least three times using different samples to ensure the relative errors were within ±3%.

## Figures and Tables

**Table 1:** Power law model fitted parameters.

Concentration of Inulin (wt%)	K (Pa-s^n^)	n	R^2^
25	0.14	0.83	0.99
27.5	70.1	0.35	0.99
30	224.6	0.33	0.99
32.5	1017	0.25	0.99
35	2448	0.24	0.99
37.5	13,626	0.07	0.99
40	41,732	0.06	0.99

## Data Availability

The data presented in this study are openly available in article.
